# Effect of the Selective Dry Cow Therapy on Udder Health and Milk Microbiota

**DOI:** 10.3390/antibiotics12081259

**Published:** 2023-07-31

**Authors:** Laura Filippone Pavesi, Claudia Pollera, Giulia Sala, Paola Cremonesi, Valentina Monistero, Filippo Biscarini, Valerio Bronzo

**Affiliations:** 1 Department of Veterinary Medicine and Animal Science, University of Milan, Via dell’Università 6, 26900 Lodi, Italy; laura.filippone@unimi.it (L.F.P.); claudia.pollera@unimi.it (C.P.); valentina.monistero@unimi.it (V.M.); valerio.bronzo@unimi.it (V.B.); 2 Department of Veterinary Science, University of Pisa, Via Livornese (SP-22), 56124 Pisa, Italy; 3 Institute of Agricultural Biology and Biotechnology, National Research Council (CNR), Via Bassini 15, 20133 Milan, Italy; paola.cremonesi@ibba.cnr.it (P.C.); filippo.biscarini@cnr.it (F.B.); 4 Laboratory of Animal Infectious Diseases—MiLab, University of Milan, Via dell’Università 6, 26900 Lodi, Italy

**Keywords:** cattle, selective dry cow therapy, milk microbiota, one health approach

## Abstract

Recently, the use of antimicrobials on dairy farms has been significantly limited from both the legislative and consumer points of view. This study aims to check the efficacy of selective dry cow therapy (SDCT) versus blanket dry cow therapy (BDCT) on bovine udder in healthy animals. SDTC is when an antibiotic is administered only to infected cows, compared with BDCT, where all cows receive an antimicrobial, regardless of their infection status. The milk samples were collected from enrolled Holstein Friesian cows 7 days before dry-off (T0) and 10 days after calving (T1) to assess somatic cell count (SCC), intramammary infections (IMIs), and milk microbiota variation. After pre-drying sampling, cows are randomly assigned to the following treatments: internal teat sealant alone (ITS; 24 cows), which is a treatment in a cow that does not receive antibiotics in SDTC, or in combination with intramammary antibiotic treatment (A+ITS; 22 cows). Non-statistically significant results are found between the two treatment groups at T1 for SCC, milk yield, and alpha diversity in milk microbiota. A statistically (*p* < 0.033) T1 IMI decrease is reported in the A+ITS group, and a significant beta diversity analysis is shown between the two timepoints (*p* = 0.009). This study confirms the possibility of selective drying without new IMI risk or increased SCC at calving, considering healthy cows without contagious infections and SCC values >200,000 cells/mL in the previous lactation.

## 1. Introduction

Mastitis is the most common and costly disease in dairy cattle worldwide. It is an inflammation of the mammary gland, and it is often caused by an intramammary infection (IMI) [1]. The risk of IMI is higher immediately after calving and is more frequently due to infections resulting from the dry period and the previous lactation [2,3]. Mastitis can appear in clinical or subclinical forms. The first one is mostly caused by environmental *Enterobacteriaceae* and *Streptococci*, particularly *E. coli* and *S. uberis* [4], while contagious agents, *Staph. aureus* and *S. agalactiae*, are mainly related to subclinical infections [5,6].

A crucial time to preserve udder health is the dry period, when the udder tissue undergoes a physiological involution characterized by an increased secretion of antibacterial substances, like lactoferrin and lysozyme [7]. These antibacterial components are part of the innate immune response and represent the predominant udder defense [8], making the mammary environment adverse to bacterial growth.

At dry-off, another natural defense mechanism against IMI is the formation of a keratin plug in the teat canal, physically preventing bacterial entry into the udder [9]. However, the keratin plug can have a delayed formation, and teats can remain open up to 10 d after drying off [10]. Internal teat sealants (ITS) have been developed to ensure the appropriate closure of the teat canal [11,12]. Moreover, a prophylactic intramammary antibiotic treatment can be introduced at the dry-off if the physiological antibacterial features are lacking [13].

In the past, blanket dry cow therapy (BDCT), consisting of the prophylactic treatment of all quarters with long-acting antimicrobials at dry-off, was widely used by dairy farmers [14]. As antibiotic use has received increasing public attention and antimicrobial resistance (AMR) has become a public health concern, efforts to reduce the antimicrobial use on dairy farms are needed. As mastitis in dairy cows is mainly caused by bacteria, antibiotics are essential for therapeutic approaches and control programs [15]. Due to the overuse of antibiotics, resistant bacterial strains have been isolated in bovine mastitis in recent years [16,17]. The phenomenon of antimicrobial resistance associated with mastitis pathogens has two main aspects. The first is related to a low recovery rate after the treatment of clinical cases, and the second is the potential transmission of resistant microorganisms and antibiotics residues to humans through dairy food consumption [18,19,20]. Selective dry cow therapy (SDTC), consisting of a specific program to avoid treating all cows with antimicrobials at dry-off, may be an alternative to BDTC [17,21,22]. Several field trials conducted in North America and Europe have investigated the effects of different approaches to selective dry cow therapy (SDCT) on antibiotic use at dry-off. These studies have consistently demonstrated significant reductions in antibiotic usage. For instance, Kabera and colleagues [23] reported a reduction of 58%, McParland and colleagues [24] (2019) found a reduction of 48%, Vasquez and colleagues [25] observed a reduction of 60%, Cameron and colleagues [26] reported a reduction of 21%, and Scherpenzeel and colleagues [27] found an impressive reduction of 85%. Additionally, Rowe and colleagues [28] found that SDCT can effectively reduce antibiotic use at dry-off without any adverse effects on intramammary infection (IMI). In the Netherlands, SDCT has been mandatory since 2012, when the preventive use of antibiotics was forbidden. This procedure has been mandatory in Italy and other European countries since January 2022, when European regulation (EU Reg 6/2019) banned the prophylactic use of antibiotics in livestock [29]. In addition, the use of some antimicrobial classes with critical importance to human health, including fluoroquinolones, macrolides, and third- and fourth-generation cephalosporins, is strictly limited in animals by the WHO *List of Critically Important Antimicrobials for Human Medicine* and EMAs categorization of antibiotics [30,31].

Several previous studies described the effects of antibiotic treatment on milk microbiota in both healthy and infected udders [32,33,34] and the effect of the presence of antibiotic residues in milk used for calf feeding [35]. Only a few studies were focused on the milk and colostrum microbiota in association with SDCT [28,36,37]. Bonsaglia and colleagues [36] analyzed changes in the milk microbiome in cows treated with a third-generation cephalosporin at dry-off, finding that drying healthy cows without antibiotics had no negative effects. Similar results were obtained by Vasquez and colleagues [25] and Biscarini and colleagues [37], investigating colostrum microbiota in cows dried with teat sealant and two different drugs (cephalonium dihydrate and benzathine cloxacillin).

Information regarding the effect of SDCT on the milk microbiota is limited, so the purpose of the present study is to evaluate the effects of SDCT versus BDCT in healthy cows through changes in milk yield, composition, microbiology, somatic cell count, and milk microbiome in a well-managed Italian dairy farm.

## 2. Results

### 2.1. Descriptive Statistics

During the experimental period, 38.6% (*n* = 58) of a total of 150 cows dried off were eligible for the study, according to the milk recording thresholds set by ARAL (Associazione Regionale Allevatori Lombardia), the dairy herd improvement association in Lombardy (Italy). Of the selected 58 cows, 12 met the inclusion criteria but were excluded after bacteriological analysis due to the isolation of major pathogens, such as *S. uberis* and *S. dysgalactiae,* or too high SCC values and isolation of non-*aureus* staphylococci (NAS). Therefore, 46 cows were enrolled in this study. Of them, 24 cows were randomized and treated only with internal sealant (ITS), and 22 cows were treated with both internal sealant and antibiotics (ITS+A). During the lactation, after the dry-period examination, no clinical mastitis was observed in the first 100 DIM in both groups.

Table 1 summarizes the main characteristics of the 2 experimental groups 7 days before dry-off (T0) and 10 days after calving (T1). The two groups were comparable for milk yield (MY) and SCC data taken from DHI controls.

### 2.2. Somatic Cell Count and IMI Evaluation

From the ITS group, most quarters were bacteriologically negative (77% at T0, 70% at T1), and NAS, including *S. sciuri*, were isolated from 21% and 27% of quarters at T0 at T1, respectively. Similarly, most quarters of the ITS+A group were bacteriologically negative at both timepoints (62% at T0, 78% at T1), and NAS, including *S. sciuri*, were isolated from 35% and 18% of quarters at T0 and T1, respectively. Environmental *Streptococci*, *S. dysgalactiae*, and *S. uberis* were isolated from 3% of quarters. *Bacillus* spp. and *Corynebacterium* spp. were isolated from lower than 3% of samples in both treatment groups and both timepoints. The χ2test showed a non-significant increase in IMI at T1 sampling in the ITS group and a statistical reduction in the ITS+A group (*p* = 0,033). The ITS samples showed an average SCC of 83.08 × 10^3^ (±285.6) cells/mL at T0, and SCC values of 68.95 × 10^3^ (±186.9) cell/mL at T1. The ITS+A samples showed a mean SCC of 127.65 × 10^3^ (±236.4) cells/mL at T0 and SCC values of 199.65 × 10^3^ (±678.1) cells/mL at T1. The differences in SCC in both group and timepoints are not statistically significant.

### 2.3. Sequencing Metrics

Sequencing the V3–V4 regions of the bacterial 16S rRNA-gene produced a total of 11,748,498 reads (joined R1–R2 paired-end reads), with an average of 127,701.1 reads per sample (46 cows × 2 time-points = 92 samples). After quality filtering, 2,214,703 sequences were removed, leaving 9,533,795 sequences for subsequent analyses (81% average retention rate, maximum 97%, minimum 36%). The number of reads for each group (ITS and ITS+A) was not significantly different at dry-off and 10 days after calving.

### 2.4. Milk Microbiota

**Figure 1:**
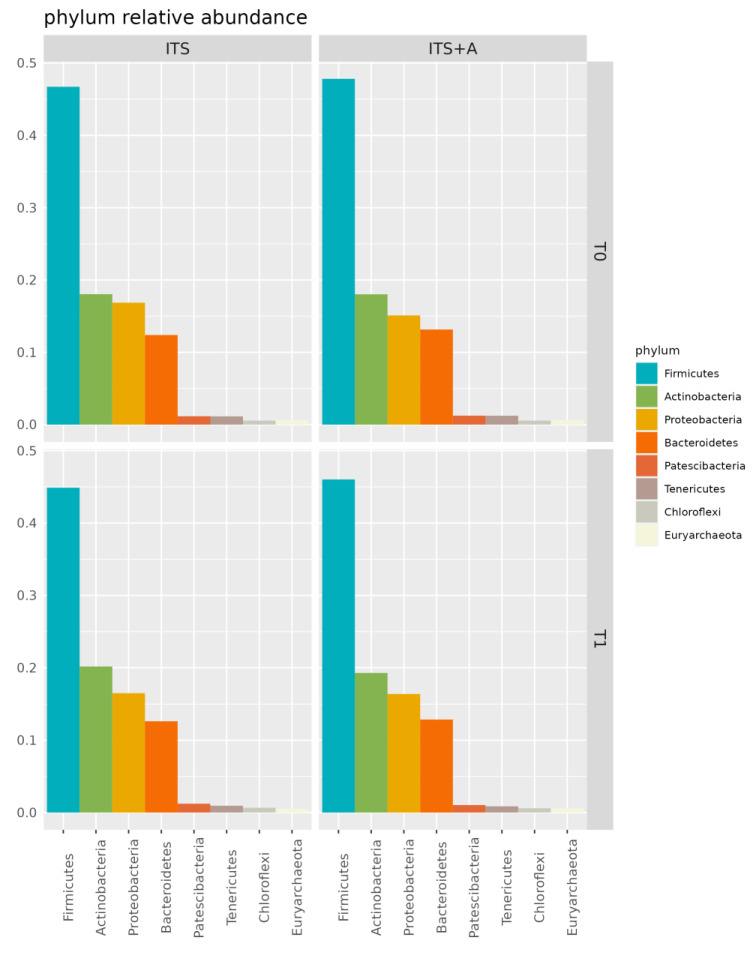
Bar plot of phylum relative abundances in the dairy cow milk microbiome over time, per treatment. Only phyla with overall relative abundance >0.5% are included.

Figure 1 shows the relative abundance of phyla in the milk microbiome overall and over time (T0 and T1). At dry-off, Firmicutes were found to be the major phylum in the milk microbiome (47% and 46% in ITS and in ITS+A, respectively), with 18% of Actinobacteria in both groups. The same phyla were the dominant in the milk microbiome in T1 sampling, with 46% and 44% of Firmicutes in ITS and in ITS+A groups, respectively, and 19% and 20% of Actinobacteria in ITS and ITS+A groups, respectively. The third and fourth most abundant phyla were Proteobacteria and Bacteroidetes at all timepoints. Both phyla remained stable in the ITS+A group (16% and 12%, respectively). In ITS T1 samples, Proteobacteria increased (15% and 16% at T0 and T1, respectively), while Bacteroidetes decreased (13% and 12% at T0 and T1, respectively). The differences were not significant between the two treatments and the two timepoints.

**Figure 2:**
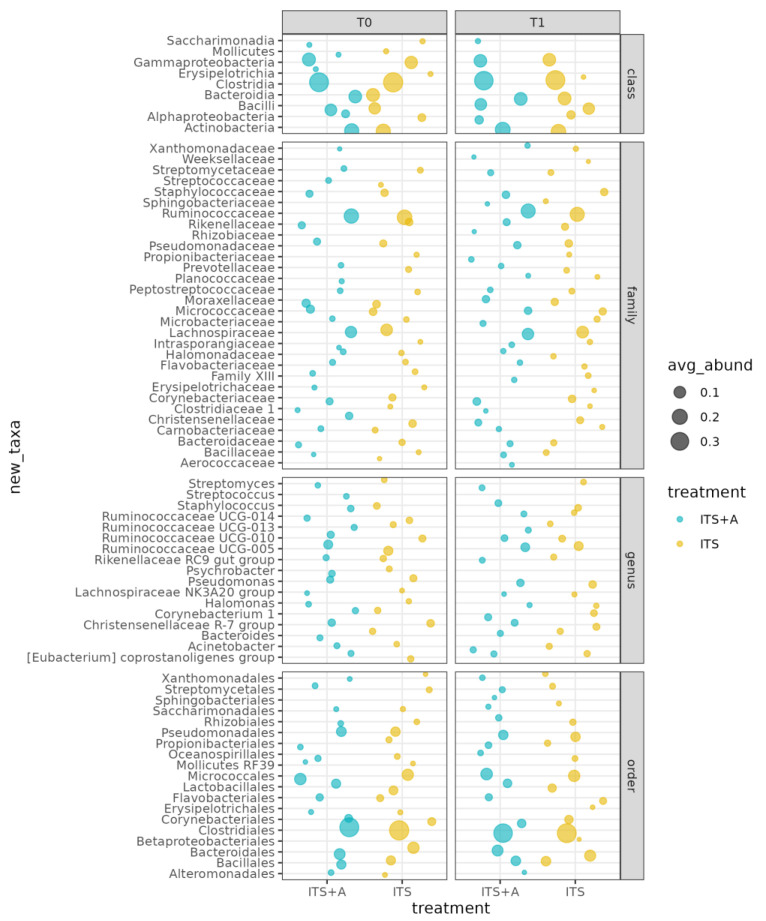
Bubble plot of the average relative abundances per class, order, family, and genus. Only taxa with average relative abundance ≥1%.

The main classes were Clostridia, Actinobacteria, Gammabacteria, Bacteroidia, Bacilli, and Alphaproteobacteria. Among them, Clostridia decreased while Bacilli and Actinobacteria increased in both groups, with differences not significant between the two treatments and the two timepoints (*p*-value > 0.05). The predominant orders were Clostridiales, Micrococcales, Bacteroidales, Pseudomonadales, and Bacillales, while the most common families were Lachnospiraceae, Moraxellaceae, Micrococcaceae, Christensellaceae, Staphylococcaceae, Corynebacteriaceae, Pseudomonaceae, and Ruminococcaceae. Figure 2 shows that the milk microbiome was dominated by the following genera: *Ruminococcus UCG-005*, *Pseudomonas*, *Christensenllacea R-7* group, *Ruminococcus UCG-010, Staphylococcus*, *Corynebacterium*, and *Acinetobacter*. The differences between the two treatments and the two timepoints were not significant (*p*-value > 0.05). 

**Figure 3:**
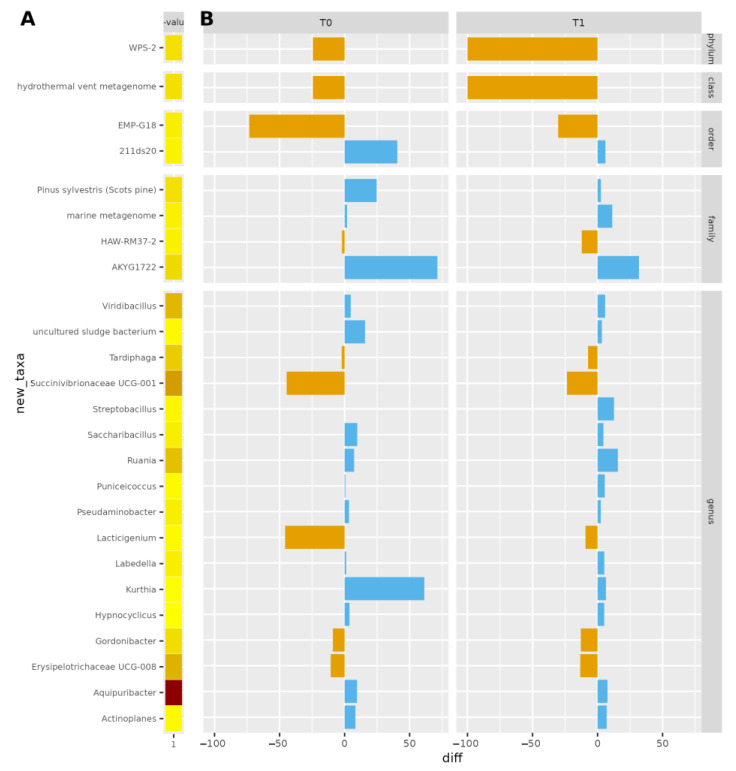
Bar plot of significantly different OTUs between treatments *p* < 0.05, at dry-off and after calving. In (**A**), the *p*-value is reported: darker colors correspond to lower *p*-values (higher significance). In (**B**), the antibiotic-teat sealant difference in terms of OTU counts is reported. Blue/orange bars indicate the differences in normalized microbial counts between ITS+A and ITS, positive (blue) or negative (orange).

The most significantly different OTUs were the genera *Aquipuribacter*, *Erysipelotrichaceae UCG-008*, *Succinivibrionaceae UCG-001*, *Ruania*, *Viridibacillus*, *Tardiphaga*, *Puniceicoccus*, and *Kurthia*. The differences between the treatments were evaluated using a linear model (ANOVA) that included the effect of timepoint and treatments nested within the cow. These data are shown in Figure 3. 

Each alpha diversity index was tested for the two timepoints, dry-off (T0) and post-partum (T1), and for the two treatment groups (ITS and ITS+A). These data are reported in Table 2. No significant differences were observed for OTU richness and alpha diversity indexes, represented by the mean Chao1 richness index. The mean Shannon richness and diversity indexes for each treatment at T0 and T1 were also not different. F:B ratio both for timepoints and treatment groups is lower than 10, with the lowest value of 3.7 for ITS at T1 and the highest value of 3.89 for ITS+A at T0.

**Figure 4:**
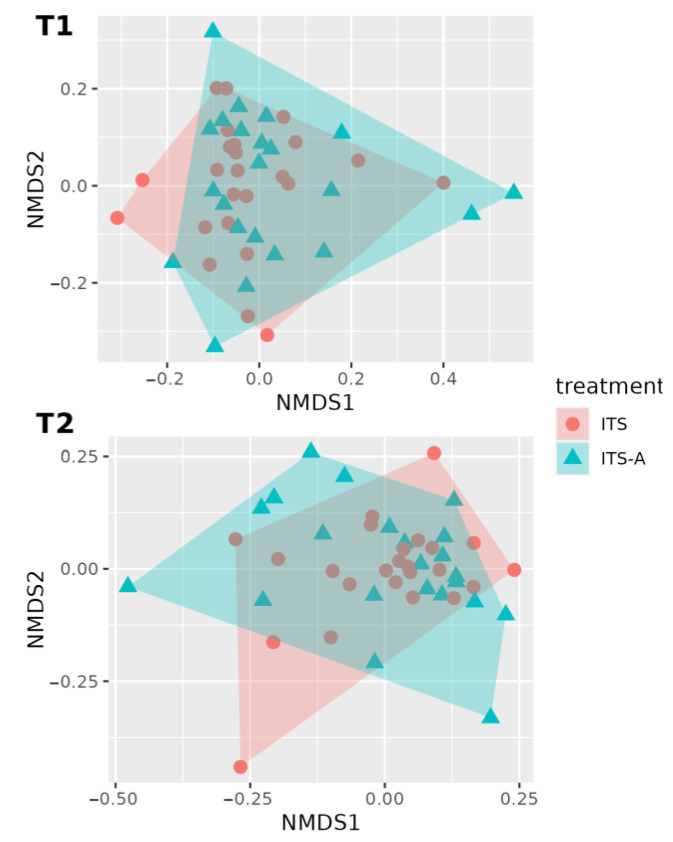
First two dimensions from the (non-metric) multi-dimensional scaling of the Bray–Curtis dissimilarity matrix. Samples were grouped by treatment within timepoint: before dry-off (T1) above, 10 days after calving (T2), below. From PERMANOVA (999 permutations): there were not any statistically significant differences between treatment (*p*-value = 0.824) and between the timepoint–treatment interaction (*p*-value = 0.812). A statistically significant difference was detected for the analysis of beta diversity for the timepoints with a *p*-value = 0.0092955.

Figure 4 shows the beta diversity analysis with PERMANOVA (999 permutations), showing no significant differences between the two treatment groups (*p* = 0.8239, SD ± 0.0113418) and, similarly, between timepoints and treatments (*p* = 0.81333, SD ± 0.0085676). A statistical difference was detected for the beta diversity analysis between the two timepoints (*p* = 0.0092955, SD ± 0.0124828).

## 3. Discussion

There has been a growing concern about the prophylactic use of antibiotics in livestock, including BDCT in cattle, and the consequent AMR emergence [38]. Recently, AMR has been rapidly spread around the world, threatening human and animal health [39]. Several studies showed that the total amount of post-partum new IMIs detected in cows treated with teat sealant alone and combined with antimicrobials at dry-off was not different [26,40]. In addition, the physical barrier resulting from the ITS use, which mimics the functions of the keratin plug [10], can support the maintenance of a favorable udder environment for commensal pathogens, maintaining the microbiome stability during the dry period [36]. Our study aimed to investigate the effect of the SDCT, mandatory for healthy cows, to assess the impact on milk yield, SCC, IMI prevalence, and milk microbiome. Specifically, cloxacillin was tested as an antimicrobial combined with ITS against ITS applied alone to support a non-antimicrobial alternative treatment to prevent new IMIs during the dry period.

The findings of this study emphasize that, in line with current European regulations, antibiotic treatment at dry-off can be avoided in healthy cows. The comparison of cows treated with selective dry cow therapy (SDCT) or blanket dry cow therapy (BDCT) in our study revealed similar milk yield during the first 100 days in milk (DIM). Differences observed in somatic cell counts (SCCs) were attributed to randomization and normal physiological variations. These results are consistent with the existing literature, although the specific criteria for selective dry-off varied across studies. Indeed, numerous studies have shown that SDCT does not impact milk yield or SCC levels in healthy cows [21,26,28,40,41].

Regarding IMI, a statistically significant reduction was observed between T0 and T1 in the group receiving antibiotic treatment. This outcome is expected since antibiotics effectively cure the IMI present at dry-off [28,42,43]. Interestingly, the group receiving only the sealant did not show a statistically significant increase in IMI. Furthermore, the isolated pathogens are categorized as minor in the literature, meaning they have a limited impact on udder health [44]. Although the major pathogens were not present at dry-off due to inclusion criteria, it is worth noting that at T1, three animals in the ITS+A group showed infection by *S. uberis*, which is considered a major pathogen [44]. Our study suggests that in healthy cows, antibiotic use at dry-off does not completely eliminate the risk of IMI in subsequent lactation. However, previous research has shown that antibiotic treatment at dry-off reduces the incidence of both past and new IMI in cows with existing IMI [28,42,43]. Importantly, no cases of clinical mastitis occurred during the first 100 DIM in our study, indicating that the use of sealant alone may be sufficient to prevent clinical mastitis in post-partum cows without prior mammary disease. This finding is consistent with the studies conducted by Cameron et al. [26] and Bradley et al. [42].

About the milk microbiota, in our study, the most abundant phyla were Firmicutes, followed by Actinobacteria, Bacteroidetes, and Proteobacteria in both treatment groups, which is in line with previous findings [36,37,45,46]. Bovine milk microbiota is prevalently composed of Firmicutes, Bacteroidetes, Proteobacteria, and Actinobacteria, which constitute the core milk microbiome. Among them, the phylum Firmicutes is typically the dominant one in the dairy healthy cow [47], including Staphylococcus, Lactobacillus, Lactococcus, Streptococcus, and Ruminococcus genera [37], the animals involved in this study. In this study, there were no differences in terms of indices and Firmicutes:Bacteroidetes (F:B) ratio between the treatment groups and in terms of relative abundances among phyla. The F:B ratio is widely accepted to have an important influence in maintaining normal intestinal homeostasis [48]. An increased or decreased F:B ratio is related to dysbiosis [48]. In cows, the gut F:B ratio is related to marked ruminal microbiota disruption and increased systemic inflammation [49]. Based on these literatures, we speculated that F:B ratio could be considered the udder health index, and the absence of difference in the two treatment groups for this parameter supports the fact that sealant alone can be effective in healthy cows at dry-off.

No differences were found in terms of alpha and beta diversity, and OTU abundance between groups, with only 25 OTUs significantly different between treatments. Derakhshani and collaborators [50], and Bonsaglia and collaborators [36] found no significant differences in alpha diversity indices before and after dry cow therapy. Moreover, they showed no differences in the milk microbiota between treatment with ceftiofur plus ITS and ITS alone. The difference highlighted in beta diversity at the drying off and after calving was according to the literature [50] and was also reported in human milk microbiota, from colostrum to late lactation [51,52,53]. The dominant families and genera were related to *Corynebacterium*, *Lachnospiraceae*, *Ruminococcaceae*, *Pseudomonas*, and *Staphylococcus*, as milk microbiota is highly affected by ruminal microbiota [46,54]. Some significant OTUs differed from findings presented in the literature, but this may be explained by the fact that farm geography, hygiene characteristics, and individual variability may impact the milk microbiota [55]. The most abundant ones have already been described in the literature. Furthermore, most of the OTUs genera decreased between T0 and T1 in the ITS+A group, and this can be due to the efficacy of the used antibiotic against most Gram-positive cocci, inhibiting β-lactamase-producing staphylococci [43]. The antimicrobial treatment did not markedly reduce the milk microbiome diversity, as shown by alpha diversity indices and beta diversity data. This result was in line with Biscarini and collaborators [37], using a similar antimicrobial molecule. This outcome could be related to the antimicrobial category, being targeted at specific pathogens and not active against the other milk microbiome. In other studies [35,36] using different antibiotic molecules with broad-spectrum activity against Gram-positive and Gram-negative bacteria, there was a reduction in milk microbiome diversity, particularly in staphylococci genera. In our work, *Staphyloccocaceae* and *Corynebacteriaceae* increased in the ITS+A group but not in ITS groups after calving, and these bacteria can easily contaminate milk samples because they are present in the teat canal and on the skin [56,57].

Our study, to the authors’ knowledge, is the first study to investigate the milk microbiota using an SDCT approach. However, additional studies that include larger sample sizes and are conducted in multiple herds are required to implement the knowledge of microbiota variations during the dry period.

Based on the findings related to udder health and microbiota, our study may have practical implications. These data strongly indicate that the adoption of SDCT serves as an effective approach to minimizing antibiotic usage while maintaining animal health and welfare. This approach offers undeniable benefits from both economic and one-health perspectives.

## 4. Materials and Methods

### 4.1. Experimental Design, Housing, Sampling, and Enrollment Criteria

A randomized controlled study on SDCT was conducted on a dairy farm located in Northern Italy from October 2020 to September 2021. The farm was selected for the absence of contagious mastitis pathogens. The Holstein Frisian herd was composed of 460 lactating cows, 98 dry cows, and 212 pregnant heifers. The herd was accredited IBR-free, immunized for neonatal diarrhea pathogens and for BVDV, digitally managed using AfiFarm 5.3 software (AfiMilk Ltd., Kibbutz Afikim, 1514800, Israel) and under a DHIA (Dairy Herd Improvement Association) program.

**Figure 5:**
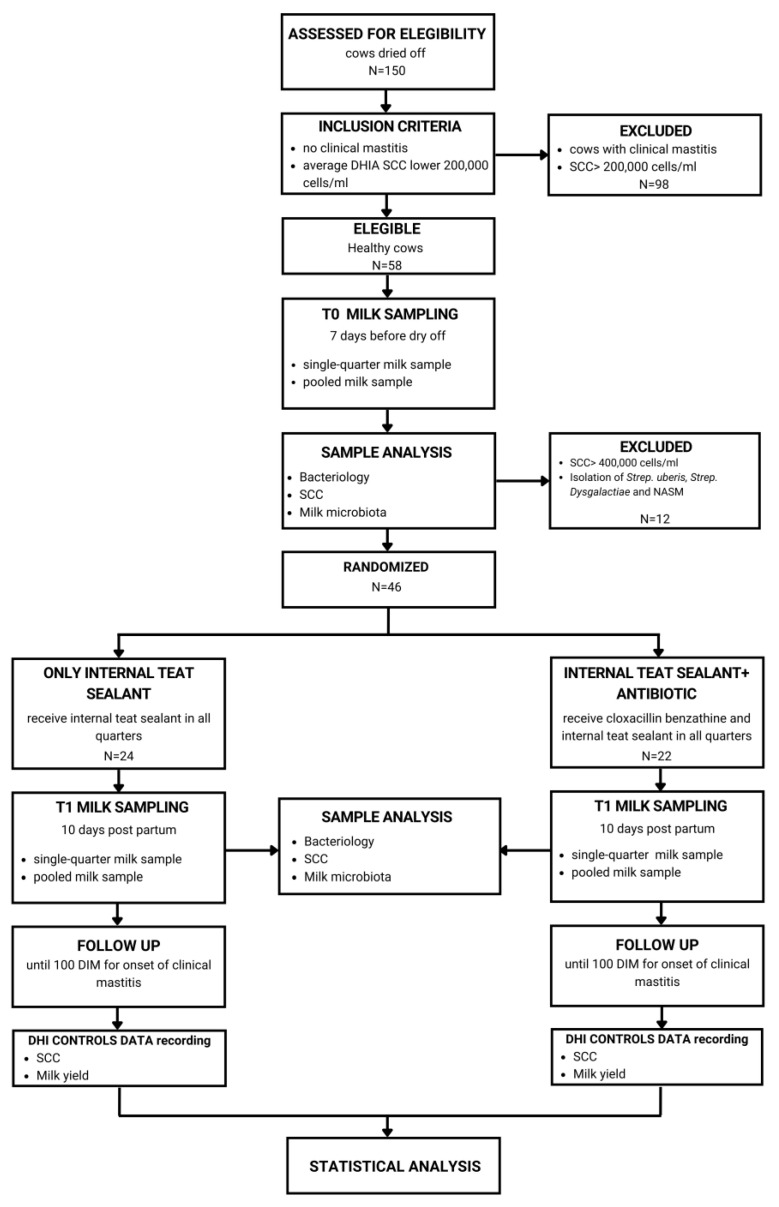
The study design. Inclusion criteria were no clinical mastitis and an average SCC lower than 200,00 cells/mL taken from dairy herd improvement (DHI) data. Eligible cows for these criteria were milk sampled seven days before dry-off (T0) in order to detect the absence of major pathogens causing mastitis and be definitely enrolled in the study. Enrolled cows were randomly assigned to the dry-off treatment group, with internal teat sealant alone and combined with antibiotics. Ten days post-partum (T1), a second milk sample was collected, and cows were monitored until 100 days in milk (DIM) for the onset of clinical mastitis.

Milking cows were housed separately from the rest of the herd in a large free stall with a slatted concrete floor and cubicles covered with soft mattresses, while dried-off cows were housed in a large free stall with straw bedding for an average of 60 days. Pregnant heifers were moved to dry-off pen four weeks before the expected date of calving. Selection criteria of cows, as shown in Figure 5, were (I) no clinical mastitis during current lactation and (II) an average SCC value lower than 200,000 cells/mL during the whole lactation, taken from DHIA controls. Seven days before dry period (T0), aseptic individual-quarter samples for IMI and SCC, and pooled milk samples for milk microbiota were collected from all cows complying with the pre-enrollment criteria, following the National Mastitis Council (NMC) guidelines [54]. If a cow showed an SCC value higher than 400,000 cells/mL and/or IMI from major pathogens and/or macroscopic alteration of the udder at T0, it would be excluded from the study. After pre-milking teat disinfection, and discarding the first streams of foremilk, approximately 10 mL of milk from each quarter and 40 mL of pooled milk from each cow were collected into sterile vials. Samples were immediately chilled on ice and transported directly to the Infectious Diseases Laboratory (MiLab. Via dell’università 6, 26900, Lodi, Italy) of the University of Milan for bacteriological analysis described below.

Each enrolled cow was randomly allocated to one of two treatment groups, receiving ITS (Easiseal, Continental Farmaceutica) alone or combined with an intramammary infusion of 500 mg of cloxacillin (Orbenin extra, Zoetis). Based on European regulations, we used semisynthetic penicillin for the SDCT in order to reduce the use of some antimicrobial classes. Ten days after calving (T1), individual-quarter and pooled milk samples were aseptically collected for the same microbiological analyses, as described below. Moreover, enrolled cows were monitored for up to 100 days in milk (DIM) in order to check possible onset of clinical mastitis [28].

### 4.2. Milk Analysis

Single quarter milk sample were stored at 4 °C until microbiological analysis and SCC, carried out by MiLab following the NMC guidelines [58]. For each sample, 10 μL of milk was streaked onto blood agar plates containing 5% defibrinated sheep blood (Microbiol, Cagliari, Italy). Plates were incubated aerobically at 37 °C and evaluated after 24 and 48 h. Bacterial colonies were isolated and provisionally identified based on morphology and hemolysis patterns. Matrix-assisted laser desorption ionization-time of flight mass spectrometry (MALDI-TOF MS) was used for their species level identification [59]. Isolates were freshly cultured on blood agar plates, and cell material from an isolated colony was deposited on the target plate using a toothpick. Samples were overlaid with one μL of α-cyano-4-hydroxycinnamic acid in 50% acetonitrile with 2.5% trifluoroacetic acid (Bruker Daltonik GmbH, Bremen, Germany). The spectra were acquired with a microFlex™ mass spectrometer (Bruker Daltonik GmbH) in the positive mode. Bacterial Test Standard (Bruker Daltonik GmbH) was used for Instrument Calibration. Spectra were automatically interpreted by the database MBT Compass^®^ 4.1. A log (score) ≥ 1.7 was the threshold for genus-level identification, and a log (score) of ≥ 2.0 was the threshold for species-level identification [59]. In particular, a quarter was defined as infected with at least one colony of a contagious pathogen (*Staph. Aureus*, *S. agalactiae*, *Prototheca* spp.) or five colonies of an environmental or opportunistic microorganism. The SCC was evaluated with a Bentley Somacount 150 (Bentley Instrument, Inc., Chaska, MN, USA). The composite milk samples were stored at −20 °C until the DNA extraction.

### 4.3. 16S rRNA-Gene Sequencing and Bioinformatics Processing

The DNA was extracted from each sample using a protocol previously described in the literature [44]. DNA quality and quantity were assessed using a NanoDrop ND-1000 spectrophotometer (NanoDrop Technologies, Wilmington, DE, USA). The isolated DNA was then stored at −20 °C until use.

Bacterial DNA was amplified using the primers described in the literature [60], which target the V3-V4 hypervariable regions of the 16S rRNA gene. All PCR amplifications were performed in 25 μL volumes per sample. A total of 12.5 μL of Phusion High-Fidelity Master Mix 2× (Thermo-Fisher Scientific, Walthem, MA, USA) and 0.2 μL of each primer (100 μM) were added to 2 μL of genomic DNA (5 ng/μL). Blank controls (no DNA template added to the reaction) were also performed. A first amplification step was performed in an Applied Biosystem 2700 thermal cycler (ThermoFisher Scientific). Samples were denatured at 98 °C for 30 s, followed by 25 cycles with a denaturing step at 98 °C for 30 s, annealing at 56 °C for 1 min, and extension at 72 °C for 1 min, with a final extension at 72 °C for 7 min. Amplicons were cleaned with Agencourt AMPure XP (Beckman Coulter Inc., Brea, CA, USA), and libraries were prepared following the 16S Metagenomic Sequencing Library Preparation Protocol (Illumina, San Diego, CA, USA). The libraries obtained were quantified by real-time PCR with KAPA Library Quantification Kits (Kapa Biosystems, Inc., MA, USA), pooled in equimolar proportion, and sequenced in one MiSeq (Illumina) run with 2 × 250-base paired-end reads.

Demultiplexed paired-end reads from 16S rRNA-gene sequencing were first checked for quality using FastQC [61] for an initial assessment. Forward and reverse paired-end reads were joined into single reads using the C++ program SeqPrep [62]. After joining, reads were filtered for quality based on the following: (I) maximum three consecutive low-quality base calls (Phred < 19) allowed; (II) fraction of consecutive high-quality base calls (Phred > 19) in a read over total read length ≥ 0.75; (III) no ”N”-labeled bases (missing/uncalled) allowed. Reads that did not match all the above criteria were filtered out. All remaining reads were combined in a single FASTA file for the identification and quantification of OTUs (operational taxonomic units). Reads were aligned against the SILVA closed reference sequence collection release 123, with 97% cluster identity [63], applying the CD-HIT clustering algorithm [64]. A pre-defined taxonomy map of reference sequences to taxonomies was then used for taxonomic identification along the main taxa ranks down to the genus level (domain, phylum, class, order, family, genus). By counting the abundance of each OTU, the OTU table was created and then grouped at each phylogenetic level. OTUs with total counts lower than 10 in fewer than 2 samples were filtered out. All of the above steps, except the FastQC reads quality check, were performed with the QIIME 1.9 open-source bioinformatics pipeline for microbiome analysis [65]. The 16S rRNA gene sequences determined in this study were deposited in the NCBI Sequence Read Archive (SRA) database with the accession number PRJEB60426.

### 4.4. Alpha and Beta Diversity Indices

The microbial diversity of the cow milk was assessed within—(alpha diversity) and across—(beta diversity) samples. All indices (alpha and beta diversity) were estimated from the filtered OTU table normalized for uneven sequencing depth by cumulative sum scaling CSS [66]. In addition, the number of observed OTUs directly counted from the OTU table, within-sample microbial richness, diversity, and evenness were estimated using the following indices: Chao1 and ACE (abundance-based coverage estimator) for richness, Shannon, Simpson, and Fisher’s alpha for diversity [67,68,69,70,71,72] and equitability (Pielou’s J’ index) for evenness [73]. The across-sample microbiota diversity was quantified by calculating Bray–Curtis dissimilarities [74]. Among groups (treated/untreated) and pairwise Bray–Curtis dissimilarities along timepoints were evaluated non-parametrically using the permutational analysis of variance approach (999 permutations; [75]). Details on the calculation of the mentioned alpha- and beta-diversity indices can be found in Biscarini et al. [76].

### 4.5. Statistical Analysis

Sample size calculation was performed using G-Power (ver. 3.1.9.7, Heinrich-Heine-Universität, Düsseldorf, Germany). To detect the minimum number of required animals a z test two-tails difference between two proportions was applied. To achieve this was used an α error of 5% (type I) and a test power of 80% with an allocation ratio of 1. The result was minimum 22 animals for group.

The incidence of mammary infections, before dry-off and after calving, were compared by χ2 test, while the SCC and milk yield data values, since data were not normally distributed, after control of the normality of the distribution of the data by means of the Shapiro–Wilk test., were compared applying a Wilcoxon non-parametric test for paired samples, taken in account two timepoints measurements on same animals for time effect in ITS and A+ITS groups, while for treatment effect at T0 and T1 timepoints between ITS and A+ITS groups, the statistical analysis was performed using a U-Mann–Whitney test for independent samples.

The milk microbiota was sampled at two timepoints (before dry-off after calving) from the same cows; therefore, observations could not be assumed to be independent of each other but were correlated within cows over time. This was considered in the following linear model used to analyze OTU differential abundance between treatments:

y_ijkz_= μ + cow_j_ + treatment_k(j)_ + timepoint_z(j)_ + (treatment × timepoint)_kz(j)_ + e_ijkz_

where y_ijkz is the OTU count for record i from cow j with treatment z at timepoint k; μ is the intercept, cow_j is the random effect of the individual cow, treatment_z(j), and |timepoint_k(j) are the systematic effects of treatment and timepoint nested within cow_j, and e_ijkz is the residual. Var(y) = Σ + I×igma_e^2^, where Σ is a block diagonal matrix with 1s on the diagonal and the covariances sigma_ij between records within cows in the off-diagonal block elements; I is the identity matrix and sigma_e^2^ is the residual variance.

### 4.6. Software

Data were collected on a spreadsheet (Excel™ 2016), and, with regard to bacteriological and SCC analyses they were analyzed using SPSS 28.0 statistical software (IBM, SPSS, Armonk, NY, USA).

Reads from 16S rRNA-gene sequencing were processed with the QIIME 1.9 pipeline [64], used also to estimate most diversity indices. The ACE index and sample-base rarefaction were estimated using own Python (https://github.com/filippob/Rare-OTUs-ACE.git accessed on 25 July 2017) and R (https://github.com/filippob/sampleBasedRarefaction accessed on 25 July 2017) scripts. Plots were generated using the ggplot2 R package Version 3 [77]. Additional data handling and statistical analysis were performed with the R environment for statistical computing [78].

## 5. Conclusions

Data regarding milk yield, SCC values, and bacterial isolations in association with the absence of alteration of the milk microbiota alpha diversity indices and the stability of the F:B ratio among the two treatment groups for the two timepoints supported that selective dry cow therapy does not interfere with udder health. As supported by this study, the efficacy of SDTC is established in cows with a healthy udder, i.e., with <200,000 cells/mL of SCC and no previous mastitis. These two characteristics are quickly assessed by DHI controls that can be uploaded to the herd management system and thus be able to identify cows before dry-off that can be dried off without antibiotic use but with the use of internal teat sealant, as a more rational use of antimicrobials in dairy farms in a one health point of view.

## Figures and Tables

**Table 1:** Number of enrolled cows and quarter sampled. Data on average milk yield (Kg) are related to the data collected from DHI controls for the two treatment groups, internal teat sealant alone (ITS) and combined with antibiotic (ITS+A), during three months before dry-off and up to three months after calving. Data related to average somatic cell count (SCC) cells × 10^3^/mL ± standard deviation, number of quarters with bacterial isolation for non-*Aureus Staphylococci (NAS)*, *Corynebacterium* spp., *Aerococcus* spp., *Bacillus* spp., bovine mastitis major pathogens and sterile quarters are related to the milk sampling at the 2 study timepoints, 7 days before dry-off (T0) and 10 days after calving (T1), for the 2 treatment groups.

	T0		T1	
	ITS	ITS+A	ITS	ITS+A
Enrolled cows	24	22	24	22
Quarter sampled	96	88	96	88
MY (kg)	27.47 ± 4.46	28.49 ± 6.31	44.12 ± 8.46	46.7 ± 5.01
SCC (cells × 10^3^/^mL^)	82 ± 162	115 ± 267	75 ± 135	113 ± 290
N. of quarters with NAS	20	31	26	16
N. of quarters with *Corynebacterium* spp.	1	1	2	0
N. of quarters with *Aerococcus viridans*	0	1	0	0
N. of quarters with *Bacillus* spp.	1	1	1	1
N. of quarters with major pathogens	0	0	0	3
N. of quarters with no isolation	74	55	67	68
N. of quarters with SCC values < 200 × 10^3^ cells/mL	75	72	84	72
N. of quarters with SCC values ≥ 200 × 10^3^ cells/mL	21	16	12	16

**Table 2:** This table shows the alpha diversity indices referred to the two treatment groups, internal teat sealant alone (ITS) and combined with antibiotic (ITS+A), for the two samplings, before dry-off (T0) and post-partum (T1). The alpha diversity indices analyzed were Chao 1, observed Otus, ace, fisher alpha, Shannon, Simpson equitability, and simpson_e. There are also *p*-values related to treatment and timepoints for each alpha diversity index.

	T0		T1		*p*-Value	
	ITS	ITS+A	ITS	ITS+A	Treatment	Time Point
Chao 1	1.836	1.863	1.797	1.970	0.405	0.797
Observed OTUs	1.263	1.283	1.239	1.306	0.456	0.982
ACE	1.841	1.882	1.804	1.951	0.423	0.906
Fisher alpha	931	963	925	970	0.523	0.997
Shannon	9.973	10.005	9.963	10.027	0.484	0.941
Simpson	0.999	0.999	0.999	0.999	0.628	0.7521
Equitability	0.972	0.972	0.973	0.972	0.326	0.952
Simpson_e	0.678	0.680	0.685	0.673	0.167	0.941

## Data Availability

The 16S rRNA gene sequences determined in this study were deposited in the NCBI Sequence Read Archive (SRA) database at https://www.ncbi.nlm.nih.gov/bioproject/PRJEB60426/ (accessed on 25 July 2017) with the accession number PRJEB60426.
